# Comparison of the push-out bond strength of two hydraulic calcium silicate-based endodontic sealers and an epoxy resin-based sealer

**DOI:** 10.4317/jced.60920

**Published:** 2023-10-01

**Authors:** Sargon Khoury, Susana Aranda-Verdú, Alberto Casino-Alegre, Jorge Rubio-Climent, Juan-Angel Cruz-Roddriguez, Antonio Pallarés-Sabater

**Affiliations:** 1 DDS,MsC. Department of Endodontics and Restorative Dentistry, Catholic University of Valencia. Private practice, Gronau Westfalen, Germany; 2 DDS, MsC. Department of Endodontics and Restorative Dentistry, Catholic University of Valencia, Valencia, Spain; private practice, Valencia, Spain; 3 DDS, MsC, PhD. Department of Endodontics and Restorative Dentistry, Catholic University of Valencia, Valencia, Spain; private practice, Teruel, Spain; 4 DDS, MsC, PhD. Department of Endodontics and Restorative Dentistry, Catholic University of Valencia, Valencia, Spain; private practice, Alicante, Spain; 5 DDS,MsC. Department of Endodontics and Restorative Dentistry, Catholic University of Valencia. Private practice, Guadalejara, Mexico; 6 DDS, PhD. Director of the Master in Endodontics, Director of the Master in Aesthetic Dentistry, Department of Endodontics and Restorative Dentistry, Catholic University of Valencia, Valencia, Spain; private practice, Valencia, Spain

## Abstract

**Background:**

The aim of this study was to assess and compare the push-out bond strength of AH Plus Bioceramic Sealer, TotalFill BC Sealer HiFlow and epoxy resin sealer AH Plus in root canals.

**Material and Methods:**

Ninety single rooted teeth with were prepared using rotatory files, 5,25 % sodium hypochlorite and 17% ethylenediaminetetraacetic acid. The teeth were divided into three groups (n=30) and obturated using the single-cone technique with TotalFill BC Sealer HiFlow in Group 1, AH Plus Bioceramic Sealer in Group 2, and AH Plus in Group 3. Three sections (coronal, middle and apical) were obtained for each root (n=270), and the push-out bond strength was evaluated for each section using an universal testing machine. The push-out bond strength among the groups was analysed using the Welch test, while the Mann-Whitney test was used to compare resistance among the coronal, middle and apical thirds of the root.

**Results:**

Significant differences were observed between the mean push-out bond strength of the two hydraulic calcium silicate sealers and the resin-based root canal sealer (*P*> 0.05). Only Group 1 exhibited significant regional differences among the root thirds, with the apical third demonstrating significantly higher strength values compared to the middle and coronal thirds.

**Conclusions:**

Based on the present study, it can be concluded that there are differences in the push-out bond strengths between the two hydraulic calcium silicate sealers (HCSSs) and the resin-based sealer, while no significant difference was found between the two HCSSs.

** Key words:**Push-out bond strength, root canal sealer, root canal obturation, hydraulic calcium silicate cements, bioceramic sealers.

## Introduction

The aim of endodontic treatment is to eliminate microorganisms and their by-products from the root canals and prevent future reinfection. However, complete asepsis is impossible to achieve. Root canal filling is intended to obtain a hermetic seal that eradicates any coronal or apical leakage pathway (1).

Root canal sealers penetrate in the dentinal tubules, fill irregular spaces, and provide adhesion between the gutta-percha and root canal walls (2-5). The resin-based sealer AH Plus (Dentsply DeTrey GmbH, Konstanz, Germany) has long been considered the gold standard for apical sealer due to its good adhesion, high radiopacity, flowability, dimensional stability, and resistance, as well as its cost effectiveness (6,7). In recent years, hydraulic calcium silicates cements (HCSCs) also called bioceramics have been introduced as an alternative. They are composed of tricalcium and dicalcium silacate, and also include a radiopacifier, additives and an aqueous or non-aqueous vehicle (8). These bioactive materials can form a direct chemical bond with the bone or even the soft tissue and induce biological changes in their environment (9). HCSCs require moisture in the dentinal tubules for setting. The hydration of silicates produces hydrated calcium silicate gel and calcium hydroxide, which react with phosphate ions, resulting in hydroxyapatite (HA) and water. The water continues to react with the remaining calcium silicates in the sealer. When the saturation of the medium is adequate, HA precipitates (9). Hence, bioactivity of the sealer refers to its ability to create an HA layer when in contact with tissue fluid, which contributes to the biocompatibility, osteoinductive and osteoconductive, and sealing properties of the material (10,11). Adequate adhesion ability of sealers to dentin is necessary to prevent bacterial leakage and endodontic failure (5). It is assumed that the force provided through occlusal loads can generate separation between the obturation material and dentin (12). Push-out or extrusion studies are used to quantify the strength of the sealer/tooth tissue interface and effectively evaluate the bond strength as fractures occur parallel to the dentin-bonding interface (13-16). TotalFill® BC Sealer HiFlow (FKG Dentaire, La Chaux-de-Fonds, Switzerland) is a hydraulic calcium silicate sealer (HCSS) that was produced to withstand high temperatures and be used with warm obturation techniques (17,18). Its predecessor, TotalFill® BC, shows excellent bond strength to radicular walls (19). However, no studies have been conducted to evaluate the bond strengths of the TotalFill BC Sealer HiFlow and a recently introduced HCSS, AH Plus Bioceramic Sealer (Dentsply Sirona, Johnson City, USA).

The aim of this study was to compare the push-out bond strength of TotalFill BC Sealer HiFlow, AH Plus Bioceramic Sealer and AH Plus in different root thirds of extracted teeth. The null hypothesis states there was no statistically significant differences between push-out bond strength values between sealers.

## Material and Methods

Ninety freshly extracted single-rooted teeth were selected and stored in a saline solution until needed. Only permanent straight single rooted teeth, upper central and lateral incisors and upper and lower canines were chosen. Preoperative buccolingual/palatal and mesiodistal radiographs were obtained to verify that the teeth only presented one root canal and the absence of previous root canal treatment, resorption, underdeveloped roots, and calcification. Cavity access was achieved using round diamond burs (Diatech; Coltene Whaledent, Altstatten, Switzerland) with a high-speed handpiece. Cavity preparation was performed using a nonactive tip bur. A size 10 K-file (Dentsply Maillefer, Ballaigues, Switzerland) was placed in the canal until it was visible at the apical foramen. The working length was determined by subtracting 1 mm from the glide path value. A glide path was established by manual instrumentation with 10, 15, and 20 K-files (Dentsply Maillefer, Ballaigues, Switzerland). The roots were then instrumented using a Protaper Gold rotary system (Dentsply Maillefer, Ballaigues, Switzerland) according to the producer´s instructions, from size S1 to F3. After each file was used, the canal was irrigated with 5.25% sodium hypochlorite (NaOCl) solution with needle and syringe (Monojet). The final irrigation protocol was performed for a minute with 5 mL of 5.25% NaOCl, 3 mL of 17% ethylenediaminetetraacetic acid (EDTA) for 3 minutes to remove the smear layer, 1 minute with 5 ml of 5.25% NaOCL and a final rinse of 10 ml of saline solution (20). Finally, the samples were dried using Protaper Gold paper points F3 (Dentsply Maillefer, Ballaigues, Switzerland). The teeth were divided into three experimental groups (n=30). The root canals were obturated with:

Group 1: TotalFill BC Sealer HiFlow (FKG Dentaire, La Chaux-de-Fonds, Switzerland).

 Group 2: AH Plus BioCeramic Sealer (Dentsply Sirona, Johnson City, USA).

Group 3: AH Plus (Dentsply DeTrey GmbH, Konstanz, Germany).

In all groups, obturation was performed using the single-cone technique with Protaper Gold F3 gutta-percha cones (Dentsply Maillefer, Ballaigues, Switzerland) and the corresponding root canal sealer. Coronal sealing was made with a flowable composite TPH Spectra ST flow (aTM, Dentsply-Caulk, Milford, DE). Each crown was sectioned using a diamond disc (KG Soresen, Barueri, SP, Brazil) mounted on a straight hand piece with water cooling at 13 mm from the apex to ensure uniform length of each tooth. The teeth were then placed in an incubator for 2 weeks at 100% humidity and 37°C to allow complete setting of the sealer. Each specimen was then sectioned perpendicular to the longitudinal thickness of 1 ± 0.1 mm in the apical, middle, and coronal thirds. Each section of each root specimen was measured using a digital calliper.

The push-bond test was conducted by loading each sample on an universal testing machine (AGS-5kNX, Shimadzu, Japan) with a 1-mm or 2-mm diameter cylindrical plunger for the coronal specimens, a 0.50-mm diameter plunger for the middle specimens, and a 0.30-mm diameter plunger for the apical specimens. The plunger only contacted the root filling during loading. The loading speed was 1 mm/min until the dislodgement of the filling material occurred. The values of the universal testing machine at that time were recorded in Newtons for each specimen. The force in Newtons was then converted into tensile strength (in MPa) (13,21). The upper and lower diameters of the specimens were measured individually, and the following formula was used: tension = force/surface area (SA). The SA was calculated using the equation “SA = π × h × (R + r)” where “R” is the mean radius of the coronal canal (mm), “r” is the mean radius of the apical canal (mm) and h is the height relative to the tapered inverted cone (mm) (13,21).

Statistical analysis of the data collected was performed using SPSS version 23 (IBM-SPSS Inc, Armonk, NY) software with a 95% confidence level. As the results of three types of sealers were to be analysed, we used the parametric Welch test for the global comparative analysis of the resistance of the three sealers and comparative analysis of the resistance between the coronal, middle and apical thirds specimens using the non-parametric Mann-Whitney Test. The results are presented through mean values, confidence intervals, and descriptive graphs.

## Results

**Figure 1:**
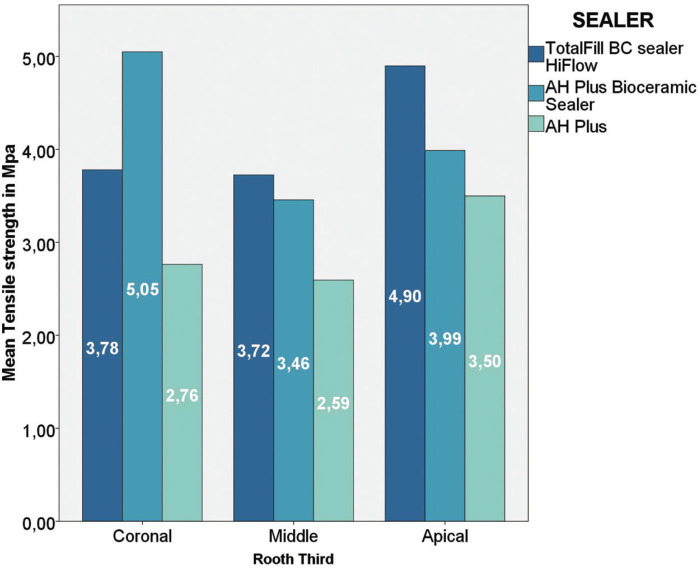
Y-axis: Force in Mpa, X-axis: The results for the different sealers separated in coronal, middle and apical thirds.

The global mean push-out bond strength values for the three root canal sealers are presented in Table 1. A significant difference was observed between the mean values of HCSSs and the resin-based root canal sealer (*P*> 0.05). The mean and standard deviation of the push-out bond strength values (in MPa) for the extrusion of the root filling material from the specimen in the coronal, middle and apical thirds specimens are shown in Figure 1. On comparing each root third between the groups, Group 2 showed statistically higher push-out bond strength values in the coronal aspect than did Group 3. In the middle and apical thirds, Group 1 showed higher push-out bond strength than did the other groups. TotalFill BC Sealer HiFlow was the only sealer that showed any significant regional differences among the root thirds. The strength values at the apical third were significantly higher than those at the middle and coronal thirds ( Table 2- Table 4).

**Table 1:**
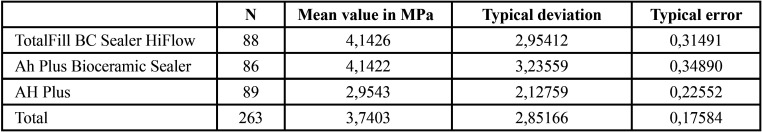
Mean and standard deviation of the push-out strength values (MPa) for the displacement of the filling material from the specimens.

**Table 2:**
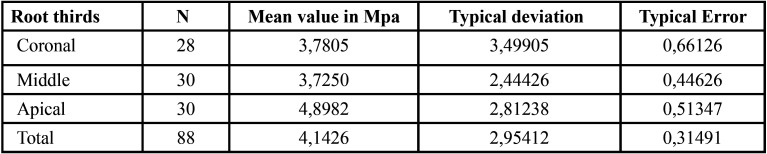
Mean and standard deviation of the push-out bond strength values (MPa) for extrusion of the root filling material from the specimen in the coronal, middle and apical thirds during the push-out test for TotalFill BC Sealer HiFlow (FKG Dentaire, La Chaux-de-Fonds, Switzerland).

**Table 3:**
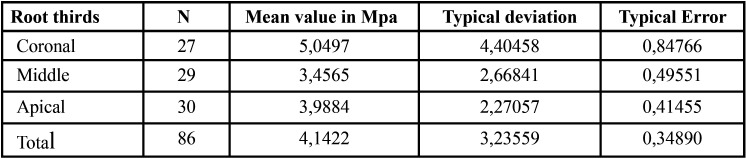
Mean and standard deviation of the push-out bond strength values (MPa) for extrusion of the root filling material from the specimen in the coronal, middle and apical thirds during the push-out test for AH Plus Bioceramic Sealer (Dentsply Sirona, Johnson City, USA).

**Table 4:**
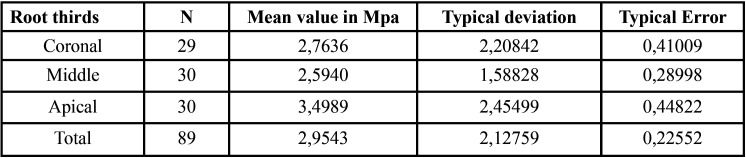
Mean and standard deviation of the push-out bond strength values (MPa) for extrusion of the root filling material from the specimen in the coronal, middle and apical thirds during the push-out test for AH Plus (Dentsply DeTrey GmbH, Konstanz, Germany).

## Discussion

This study aimed to compare the push-out bond strength of the different root canal sealer to determine if there are differences between HCSSs and resin-based sealer.

Root canal sealers undergo continual advancements for improved outcomes. The bonding capability of the endodontic sealer can affect its clinical behaviour since the sealer needs to be able to resist mechanical friction and retention (22). To evaluate bonding strength, the push-out bond model is an efficient and reliable method because it permits evaluation of regional differences in bond strength (23). It is a reproducible method as the same loading force for each sample is needed and even low values can be detected (24). It has been proposed that bonding between the root canal sealer and gutta-percha helps resist dislodgement and that chemical bonding to the root dentin increases the push-out strength needed for dislodgement (24–27). Considering these factors, the push-out bond strength values appear directly proportional to the bonding capability of the material (24,25). High strength could reduce bacterial microleakage and improves the longevity of endodontic treatment (15).

In the present study, we found no significant differences in the push-out bond strength of the two HCSSs, which might be related to their composition and similar viscosities. However, a statistically significant differences between the two HCSSs and the resin-based root canal sealer was observed. The greater bonding strength of the HCSSs in our study may be attribuTable to “alkaline caustic etching”, which involves ion exchange and penetration of the minerals of the HCSSs into the dentine, with subsequent creation of a mineral infiltration zone at the interface. This zone reduces space formation in the canal as compared to that by an AH Plus sealer (28-30). Although the compositions of the two HCSSs according to the manufacturer are not the same, both contain zirconium oxide and tricalcium silicate as main components. The AH Plus Bioceramic Sealer contains more zirconium oxide (50-70%) than the TotalFill BC Sealer HiFlow (35-45%). Further, the TotalFill BC Sealer HiFlow contains more tri-calcium silicate (20-35%) than the AH Plus Bioceramic Sealer (5-15%). Variations in the weight percentage of zirconium oxide, tri-calcium silicate and the presence of dicalcium silicate in the TotalFill BC Sealer HiFlow seem to have no effect on the push-out bond strength. As mentioned by Dewi *et al*., HCSSs present good adhesion due to their hydrophilicity and small particle size, which allows them to have good flowability and fit into anatomical structures such as dentinal tubules (19,31). In addition, the HCSSs show better adaptability and do not apply intracanal pressure during dentinal tubule penetration (32–35). This may explain the good push-out bond resistance presented by the two HCSSs.

To our knowledge, only one existing study assessed the push-out bond strength of the new HCSS of FKG (31). However, no studie on the push-out bond strength of the AH Plus Bioceramic Sealer have been published. Similar to our study, Dewi *et al*. (31) found that HCSSs such as the TotalFill BC Sealer HiFlow had higher push-out bond strengths than resin-based root canal sealers. The values for the TotalFill BC Sealer HiFlow with the single-cone obturation technique were consistent with our results, confirming the reliability of the push-out strength test. However, Dewi *et al*. used bioceramic-coated gutta-percha points according to the manufacturer’s instructions to bond to the root canal sealer and create a mono-block.

The strength of the sealers observed in our study was slightly higher than that observed by Sagsen *et al*. (13), who compared AH Plus, root SP root canal sealer (Innovative BioCreamix Inc, Vancouver, Canada), and MTA Fillapex (Angelus Soluciones Odontologicas, Londrina, Brazil). Costa *et al*. compared Epiphany (Pentron Clinical Technologies, Wallingford, CT, USA) variations with Hybrid Root SEAL sealer (Sun Medical, Tokyo, Japan) (21). Nonetheless, the differences in results can be attributed to various factors such as different irrigation protocols used by Sagsen *et al*. or storage conditions post-extraction. Other studies have compared the conventional HCSS of FKG/Brasseler to other types of root canal sealers. Ahmad *et al*. (27) demonstrated that the TotalFill BC sealer had higher push-out bond strength than AH Plus regardless of the obturation technique.

Our results, which indicated that a higher bond strength to root dentin may impact the sealer’s ability to resist disruption by micromechanical retention or friction are in accordance with other studies (22-27), which may serve as a reference for future research.

The results highlight the importance of considering the choice of root canal sealer in clinical practice. The higher push-out bond strength observed with the HCSSs suggests their potential to enhance the resistance of the sealer to disruption. This finding may have implications for the long-term success of root canal treatments.

The main limitation is that in the present study, the push-out bond strength was evaluated in a laboratory setup that allowed controlled setting of the root canal sealer. The bonding capacities of HCSSs should be further investigated in a clinical environment. Furthermore, as shown in Table 1 a few samples were lost due to values that were wither unproportionally high or low. These could be traced down to errors during the sample preparation, hence we were not able to be incorporate them into the analysis.

In conclusion, the findings of this study suggest, that the push-out bond strengths in HCSSs is higher than the resin-based root canal sealer. However, no significant difference was observed between the two HCSSs evaluated. TotalFill BC Sealer HiFlow was the sealer that showed the highest strength values at the apical third.

Investigations into their sealing ability, the effect of different obturation techniques on bond strength and the influence of root canal anatomy could provide valuable insights for future studies.

## References

[1] Segura-Egea JJ, Gould K, Şen BH, Jonasson P, Cotti E, Mazzoni A (2017). European Society of Endodontology position statement: the use of antibiotics in endodontics. International Endodontic Journal.

[2] Tyagi S, Mishra P, Tyagi P (2013). Evolution of root canal sealers: An insight story. Eur J Gen Dent.

[3] Wang Y, Liu S, Dong Y (2018). In vitro study of dentinal tubule penetration and filling quality of bioceramic sealer. PLoS One.

[4] Komabayashi T, Colmenar D, Cvach N, Bhat A, Primus C, Imai Y (2020). Comprehensive review of current endodontic sealers. Dental Materials Journal.

[5] Grossman LI (1976). Physical properties of root canal cements. J Endod.

[6] Asawaworarit W, Pinyosopon T, Kijsamanmith K (2020). Comparison of apical sealing ability of bioceramic sealer and epoxy resin-based sealer using the fluid filtration technique and scanning electron microscopy. J Dent Sci.

[7] Lee SJ, Chung J, Na HS, Park EJ, Jeon HJ, Kim HC (2013). Characteristics of novel root-end filling material using epoxy resin and Portland cement. Clin Oral Investig.

[8] Camilleri J, Atmeh A, Li X, Meschi N (2022). Present status and future directions: Hydraulic materials for endodontic use. Int Endod J.

[9] Koch KA, Brave D, Nasseh A (2010). Bioceramic technology: Closing the endo-restorative circle, Part 1. Dent Today.

[10] Trope M, Bunes A, Debelian G (2015). Root filling materials and techniques: bioceramics a new hope?. Endod Top.

[11] Jitaru S, Hodisan I, Timis L, Lucian A, Bud M (2016). The use of bioceramics in endodontics - literature review. Clujul Med.

[12] Tawil PZ, Abe D, Duggan DJ, Galicia JC (2016). MTA: A Clinical Review. Compend Contin Educ Dent.

[13] Sagsen B, Ustün Y, Demirbuga S, Pala K (2011). Push-out bond strength of two new calcium silicate-based endodontic sealers to root canal dentine. Int Endod J.

[14] Amoroso-Silva PA, Marciano MA, Guimarães BM, Duarte MAH, Sanson AF, Moraes IG de (2014). Apical adaptation, sealing ability and push-out bond strength of five root-end filling materials. Braz Oral Res.

[15] Barbizam JVB, Trope M, Tanomaru-Filho M, Teixeira ECN, Teixeira FB (2011). Bond strength of different endodontic sealers to dentin: Push-out test. J Appl Oral Sci.

[16] Moinzadeh AT, Mirmohammadi H, Hensbergen IAM, Wesselink PR, Shemesh H (2015). The correlation between fluid transport and push-out strength in root canals filled with a methacrylate-based filling material. Int Endod J.

[17] Hadis M, Camilleri J (2020). Characterization of heat resistant hydraulic sealer for warm vertical obturation. Dent Mater.

[18] Camilleri J (2020). Classification of Hydraulic Cements Used in Dentistry. Front Dent Med.

[19] Hasnain M, Bansal P, Nikhil V (2017). An in vitro comparative analysis of sealing ability of bioceramic-based, methacrylate-based, and epoxy resin-based sealers. Endodontology.

[20] Camilleri J, Atmeh A, Li X, Meschi N (2022). Present status and future directions: Hydraulic materials for endodontic use. Int Endod J.

[21] Costa JA, Rached-Júnior FA, Souza-Gabriel AE, Silva-Sousa YTC, Sousa-Neto MD (2010). Push-out strength of methacrylate resin-based sealers to root canal walls. Int Endod J.

[22] Huffman BP, Mai S, Pinna L, Weller RN, Primus CM, Gutmann JL (2009). Dislocation resistance of ProRoot Endo Sealer, a calcium silicate-based root canal sealer, from radicular dentine. Int Endod J.

[23] Gesi A, Raffaelli O, Goracci C, Pashley DH, Tay FR, Ferrari M (2005). Interfacial strength of Resilon and gutta-percha to intraradicular dentin. J Endod.

[24] Ungor M, Onay EO, Orucoglu H (2006). Push-out bond strengths: The Epiphany-Resilon endodontic obturation system compared with different pairings of Epiphany, Resilon, AH Plus and gutta-percha. Int Endod J.

[25] Shipper G, Ørstavik D, Teixeira FB, Trope M (2004). An evaluation of microbial leakage in roots filled with a thermoplastic synthetic polymer-based root canal filling material (Resilon). J Endod.

[26] Onay EO, Ungor M, Ari H, Belli S, Ogus E (2009). Push-out bond strength and SEM evaluation of new polymeric root canal fillings. Oral Surgery, Oral Med Oral Pathol Oral Radiol Endodontology.

[27] Al-Hiyasat AS, Ahmad SA (2019). The effect of obturation techniques on the push-out bond strength of a premixed bioceramic root canal sealer. J Dent.

[28] Kakoura DDS F, Pantelidou DDS, PhD O (2018). Retreatment Efficacy of Endodontic Bioceramic Sealers: A Review of the Literature. Odovtos - Int J Dent Sci.

[29] Camilleri J (2017). Will Bioceramics be the Future Root Canal Filling Materials?. Curr Oral Heal Reports.

[30] Atmeh AR, Chong EZ, Richard G, Festy F, Watson TF (2012). Dentin-cement interfacial interaction: Calcium silicates and polyalkenoates. J Dent Res.

[31] Dewi A, Upara C, Sastraruji T, Louwakul P (2022). Effect of a heat-based root canal obturation technique on push-out bond strength of the classical bioceramic and new HiFlow sealer. Aust Endod J.

[32] Jeong JW, DeGraft-Johnson A, Dorn SO, Di Fiore PM (2017). Dentinal Tubule Penetration of a Calcium Silicate-based Root Canal Sealer with Different Obturation Methods. J Endod.

[33] Zafar MS (2020). Prosthodontic applications of polymethyl methacrylate (PMMA): An update. Polymers (Basel).

[34] Eltair M, Pitchika V, Hickel R, Kühnisch J, Diegritz C (2018). Evaluation of the interface between gutta-percha and two types of sealers using scanning electron microscopy (SEM). Clin Oral Investig.

[35] Candeiro GTDM, Correia FC, Duarte MAH, Ribeiro-Siqueira DC, Gavini G (2012). Evaluation of radiopacity, pH, release of calcium ions, and flow of a bioceramic root canal sealer. J Endod.

